# Prospective longitudinal associations between adverse childhood experiences and adult mental health outcomes: a protocol for a systematic review and meta-analysis

**DOI:** 10.1186/s13643-023-02330-1

**Published:** 2023-09-30

**Authors:** Christina Thurston, Aja Louise Murray, Hannabeth Franchino-Olsen, Franziska Meinck

**Affiliations:** 1 School of Social and Political Science, University of Edinburgh, Chrystal Macmillan Building, 15a George Square, Edinburgh, EH8 9LD UK; 2 Department of Psychology, University of Edinburgh, Edinburgh, UK; 3 School of Public Health, University of the Witwatersrand, Johannesburg, South Africa; 4 Faculty of Humanities, North-West University, Potchefstroom, South Africa

**Keywords:** ACE, Longitudinal, Depression, Anxiety, Psychosis, Suicide, Post-traumatic stress, Suicide, Review, Meta-analysis, Protocol

## Abstract

**Background:**

Research cites a strong, dose–response relationship between adverse childhood experiences (ACEs) and poor adult mental health outcomes including anxiety, depression, post-traumatic stress disorder (PTSD), self-harm, suicidality, and psychotic-like experiences.

**Aim:**

To systematically investigate the existence and strength of association between ACEs and adult mental health outcomes in prospective longitudinal studies. The review will focus on the outcomes: anxiety, depression, PTSD, self-harm, suicidal ideation, and psychotic-like experiences.

**Methods:**

Twelve electronic databases will be searched: Embase, PsycINFO, MEDLINE, and Global Health through the OVID interface. ProQuest will be used to search Public Affairs Information Service (PAIS), Dissertations and Theses, Sociology Database (including Sociological Abstracts and Social Services Abstracts), PTSDpubs (formerly The Published International Literature on Traumatic Stress (PILOTS) Database) and Applied Social Sciences Index and Abstracts (ASSIA). CINAHL, World Health Organisation (WHO) Global Index Medicus, and WHO Violence Info will also be searched. Eligible studies will be double screened, assessed, and their data will be extracted. Any disagreement throughout these processes will be settled by a third reviewer. If enough studies meet the criteria and the methodological quality of each study is sufficient, a meta-analysis will be conducted.

**Analysis:**

A narrative synthesis of included studies and the associations between ACEs and adult mental health will be completed. If the number of studies included per mental health outcome is two or more, a multi-level meta-analysis will be completed using odds ratio effect sizes as outcomes.

**Discussion:**

This review will contribute to the existing body of literature supporting the long-term effects of ACEs on adult mental health. This review adds to previous reviews that have either synthesised cross-sectional associations between ACEs and mental health outcomes, synthesised longitudinal studies exploring the effect of ACEs on different physical and mental health outcomes or synthesised longitudinal studies exploring the effect of ACEs on the same mental health outcomes using different methods. This review aims to identify methodological weaknesses and knowledge gaps in current literature that can be addressed in future primary studies.

**Systematic review registration:**

This protocol has been registered in PROSPERO (CRD42021297882).

**Supplementary Information:**

The online version contains supplementary material available at 10.1186/s13643-023-02330-1.

## Background

The term “Adverse Childhood Experiences” or “ACEs” was first coined in Felitti et al.’s [1] seminal “Adverse Childhood Experiences Study” and was used to describe a group of specific childhood experiences. Adverse childhood experiences (ACEs) can broadly be defined as potentially traumatic life events occurring in the first 18 years of life [2]. Experiences that are defined as ACEs vary within the literature; however, they can broadly be categorised into three overarching classifications: abuse (emotional, sexual, and physical), neglect (emotional and physical), and household dysfunction (alcohol and/or drug abuse in the house, imprisoned family member, mother treated violently, and parental loss, separation, or divorce) [3]. While these ACEs are the most heavily researched, this list is not exhaustive. There are further experiences recognised as ACEs in research that this review will also consider, such as being bullied [4], community and collective violence [5], parental mortality and morbidity [6], child marriage, [7] and child trafficking [8].

Over the last three decades, extensive research has explored the relationship between ACEs and the later onset of poorer cognitive, emotional, and behavioural outcomes [1, 9]. Strong cross-sectional and longitudinal relationships have been established between ACEs and an increased risk of developing various psychiatric problems including depression [10], anxiety disorders [11], suicidal ideation [12] and psychosis [13]. The increasing body of extant literature has concluded that ACEs are a dangerous public health problem [14], and emerging research has recognised adult mental illness as one of the largest public financial burdens associated with ACEs [15].

### Adverse childhood experiences and poor life outcomes

In 1998, Felitti et al. conducted the aforementioned “Adverse Childhood Experiences Study” in Southern California in the United States of America. The retrospective cohort study collected data over two waves from 1995–1997 and was responded to by 17,337 participants. Participants were selected for the study from their attendance of the Kaiser Permanente’s Health Appraisal Centre (HAC) due to being adult members of the Kaiser Health Plan in San Diego County. The study was designed to explore whether there was a relationship between early life adversity and adult physical and mental ill-health. In both waves, adults who had completed a standard medical evaluation at HAC one-to-two weeks’ prior were asked about adverse childhood experiences (ACEs) and health behaviours through questionnaires sent by mail. The HAC evaluations provided standardised medical histories and formed part of the ACE study database. There were ten adverse childhood experiences included that were separated into two broader categories of childhood maltreatment and household dysfunction [16]: emotional and physical neglect; emotional, sexual, or physical abuse; living in a household where members abused substances, where there was violence against the mother, where members were mentally ill/ suicidal or where members were ever incarcerated; and parental separation or divorce.

Felitti et al. [1] found that around two-thirds of the sample experienced at least 1 ACE and around 12.5% experienced at least 4 ACEs. When exploring later negative life events, the researchers found a variety of health outcomes that were strongly associated with having 4 or more ACEs. For example, compared to having no ACEs, those with 4 or more were around 4.6 times more likely to have had depressed mood in the past year, 12.2 times more likely to have ever attempted suicide, 7.4 times more likely to consider themselves and alcoholic and 10 times more likely to have ever injected drugs. A strong dose–response relationship was established between one’s number of ACEs and poor outcomes, including the emergence of later life mental difficulties and physical diseases.

After the pioneering work of Felitti et al. [1], ACEs studies have been conducted globally that confirm ACEs are associated with a variety of poor outcomes [17]. For example, studies have evidenced the association between ACEs and suicidal behaviour in South Africa [18], heavy drinking amongst other health-harming behaviours in the United Kingdom [19], depressed affect in California, North America [20], illicit drug use in Brazil, South America [21] and anxiety, depression and PTSD symptoms in South-East Asia [22]. In recent years, ACE studies have also been synthesised in systematic reviews and meta-analyses. In their systematic review and meta-analysis, Hughes et al. [23] demonstrated the significant, deleterious effect multiple ACEs have on lifelong health. Other systematic reviews include Norman et al. [24] and Kalmakis and Chandler [25], whose results suggested significant associations between ACEs and various long-term mental health outcomes and health-harming behaviours, including depressive disorders, suicide attempts, PTSD, substance misuse, and sexual risk behaviour. Sahle and colleagues’ [26] recent umbrella review also confirmed strong, significant associations between ACEs and common mental disorders.

## Rationale

Despite the seminal ACE study [1] following the original participants to measure the emergence of poor health outcomes over time, the study still measured ACEs retrospectively. In current literature, retrospective reporting of ACEs by adults remains the most common method of obtaining comprehensive self-reports of adversity [27]. Studies using test–retest reliability to explore the consistency of reports of ACEs over time generally find stability in retrospective measures [28]. However, due to the reporting of adversity being many years after the event occurred [29], one must consider the possible biases that may result in inaccurate data. Scepticism of the validity of childhood information collected in adulthood has existed for over five decades now, as Yarrow, Campbell and Burton [30] suggested recollection of childhood information may be largely contingent on the information and narration of events told by one’s parents. Retrospective reporting of ACEs is thought to be at a far higher risk of inaccuracy than prospective reporting (the reporting of ACEs as they emerge) due to further issues such as recall bias [31], memory decay [32] and mood-congruent bias [33], where the reporting of historical events is determined by one’s current mental state. For example, researchers have posited that adults diagnosed with mental disorders such as depression exhibit specific “retrieval biases” that subsequently result in superior recall of more negative historical events and fewer positive events [34, 35].

Henry, Moffitt, Caspi, Langley and Silva [36] explored the agreement between retrospective and prospective reporting of ACEs across a prospectively studied large sample of adolescents. Several categories of information were compared and whilst more objective content such as moving house and height were consistently reported between prospective and retrospective measures, the poorest agreement was found in the more subjective information such as one’s psychological state and childhood adversities such as maternal mental illness and family conflict. The lack of agreement between retrospective and prospective reports of childhood adversities has also been substantiated in more recent research. For example, Baldwin et al.’s [37] systematic review and meta-analysis found that around 52% of participants who prospectively reported adversity in childhood did not go forward to report it retrospectively. Furthermore, 56% of participants who retrospectively disclosed ACEs had not reported this adversity prospectively. Whilst it has been argued the poor agreement between retrospective and prospective approaches to reporting is due to poor validity of the retrospective measures, there may be other reasons for such disagreement. For example, prospective measures may record ACEs before childhood ends and subsequently may not capture adverse events that happened after data collection in the way that retrospective accounts of adversity across the whole of childhood do [37]. This current systematic review has subsequently chosen to only include studies using prospective measures of ACEs in line with Baldwin et al.’s [37] recommendation not to compare studies across prospective and retrospective approaches to data collection. This is primarily due to the large discrepancy in populations they identify.

The current review will include prospective, longitudinal research designs that study ACEs instead of retrospective, cross-sectional designs due to their ability to explore temporal sequencing of events [38]. Prospective studies offer valuable information about developmental changes, incidence rates of ACEs, and a better understanding of the timing and chronicity of ACEs [39, 40]. Furthermore, without the temporal patterning of events, the direction of the relationships cannot not be established [41]. This is one of the main reasons why retrospective adult studies of ACEs are not sufficient to understand causal pathways between ACEs and adult outcomes [42]. In prospective longitudinal studies, the collection of data through time allows opportunity for confounding variables to be measured and adjusted for at each time point [43]. However, it should be acknowledged that causal mechanisms between adverse childhood experiences and later-life poor outcomes such as mental ill-health are difficult to infer- even in longitudinal research [44]. This is due to many factors including under-reporting biases in the reporting of ACEs [39] and a lack of consideration of unobservable genetic components and family characteristics that contribute to any causal relationships [44]**.** These limitations mean we do not aim to infer any causal relationships from the findings in our review. Despite the limitations of prospective longitudinal ACEs studies, prospective measures of ACEs still provide a valuable tool for identifying risk markers for later poor outcomes in adults [45]. The six mental health outcomes (depression, anxiety, PTSD, suicidal ideation, self-harm, and psychotic-like experiences) were selected as they represent six of the most commonly assessed mental health outcomes in research exploring the association between ACEs and later-life mental ill-health.

## Methods/ design

### Aim and review questions

The main aim of this systematic review and meta-analysis is to address the gap in the literature by exploring the associations between ACEs and the specific adult mental ill-health outcomes of depression, anxiety, PTSD, psychotic-like experiences, suicidality, and self-harm in prospective longitudinal research globally. A considerable portion of prospective longitudinal research focuses on the relationship between ACEs and mental health outcomes earlier in development (e.g., [46–50]). However, we are interested in exploring whether such associations between ACEs and mental health remain into adulthood and across the lifespan. There have been less syntheses of such longer-term associations, and this was a main reason we wanted to limit our review to adult mental health outcomes.

The authors are aware of a similar systematic review and meta-analysis that recently explored longitudinal associations between childhood trauma and adult mental disorder [51]. However, the current review provides the novel inclusion of grey literature, differing mental health outcomes (unlike McKay et al. [51] who included the outcomes of depression, anxiety, psychotic disorder and bipolar disorder, this study seeks to include anxiety, depression, psychotic-like experiences, PTSD, suicidality, and self-harm) and a lower threshold for the measurement of mental health outcomes. Unlike McKay et al. [51], the current study stipulates the mental health outcomes need not be formal psychiatric diagnoses using established diagnostic criteria for mental disorders in adulthood as such use of these measures is rare in low-and middle-income countries. Furthermore, this review completes an updated and more comprehensive database search (including ProQuest Dissertations and Theses comprising of grey literature), which, in turn, may reduce potential effects of algorithm or publication bias [52]. We felt that to ensure we captured a holistic overview of all literature on the topic that grey literature should be included. Grey literature is often excluded from large systematic reviews, and we feel that this may unintentionally exclude certain geographical locations that lack funding to support peer-reviewed study production and publication. The Newcastle–Ottawa Scale will still be used to appraise study quality of any grey literature found and their findings would still have to fit our stringent inclusion and exclusion criteria.

This protocol has been registered in PROSPERO (CRD42021297882) and followed the PRISMA-P (Preferred Reporting Items for Systematic Review andMeta-Analysis Protocols) 2015 statement: recommended items to address in a systematic review protocol [53] (see checklist in Additional file 1).

Certain questions may not be answered as they remain contingent on enough studies fitting the criteria. The Population-Issue-Comparison-Outcome (PICO)/ Population-Exposure-Outcome (PEO) framework [54] was used to create the overarching review question which is:

“Are adults who have been exposed to adversity in childhood at an increased risk of developing mental illness(es) compared to adults who have not been exposed to adversity in childhood?”

We will address the following sub-questions:

1. What are the associations between ACEs and depression, anxiety, PTSD, suicidal ideation, self-harm, and psychotic-like experiences in adulthood with a specific interest in the prevalence of research conducted in high-income countries versus low-and middle-income countries?
2. Which geographical locations does the evidence on ACEs stem from?
3. Which ACEs have the largest negative associations with adult mental health?
4. Is there a cumulative effect of ACEs on mental health outcomes?
5. Is the association between ACEs and adult mental ill-health moderated by geographical location of study?
6. Is the association between ACEs and adult mental ill-health moderated by peer-reviewed status?
7. Is the association between ACEs and adult mental ill-health moderated by study design or analysis?
8. Is the association between ACEs and adult mental ill-health dependent on age of onset at the first adversity?
9. What is the quality of studies looking at longitudinal associations between ACEs and mental health outcomes?

Question 4 pertains to any study that includes a measure of cumulative adversity. We aim to pool the effect sizes from analyses that have used, for example, a continuous measure of cumulative ACEs such as “0 Adversity, 1 Adversity, 2 Adversity” or a measure using the widely recognised cut-off 0–3 ACEs vs 4 + ACEs. Question 5 was created as the geographical location of studies may influence the prevalence and types of childhood adversity. For example, evidence suggests ACEs may be more common in low-resource settings/ low- and middle-income countries [55–57]. Question 6 was included as there may be publication bias present. This means the studies published in peer-reviewed journals could over-represent the significance of associations given the fact that many articles published show statistically significant results [58, 59]. Question 7 was created as analytic choices across studies may influence the results found and reported [60]. Furthermore, the effect sizes reported for the relationships between ACEs and adult mental health may vary depending on what design is used (e.g., whether the study used a self-reported, prospective measure of adversity or whether the study used data-linkage to official court records). This may be due to many reasons, including under-reporting biases and different thresholds of what events are recorded or “counted” as an adversity [61–63]. Question 8 was created as the age of onset may align with critical/ sensitive periods for the development of mental health symptoms and thus could again influence the strength of associations [64].

### Inclusion and exclusion criteria

We adopted the Population-Exposure-Outcome (PEO) model to aid in outlining the inclusion and exclusion criteria seen in Tables 1 and 2, respectively.

**Table 1:** Inclusion criteria using the PEO model where applicable

Criteria	Inclusion
Design	Studies included must be prospective panel or cohort studies and have at least 2 time points
Publication Status	Studies can be peer-reviewed or non-peer-reviewed
Location	The study can be conducted in any geographical location
Source of Evidence	Findings can be from books, research articles, government documents, conference abstracts, annual reports, dissertations, and theses
Date	The studies were published from 1990- 2021, but the data may have been collected before 1990. This specific period has been chosen as it aligns with the drafting of the United Nations Convention on the Rights of the Child by the United Nations [65]
Language	The studies are readily available in English due to limited research resources prohibiting translation
Population	The sample should be from the general human population but need not be nationally representative
Population	The sample does not need to be selected based on mental ill-health or exposure to ACEs. However, the sample can be “clinical” or “special” in the sense that they may be recruited through an outpatient clinic or attend an outpatient clinic (for example, an outpatient clinic for depression or an outpatient HIV clinic). They may also have a diagnosed mental illness or be at a higher risk of experiencing ACEs or poor mental health through, for example, coming from a disadvantaged background
Exposure	There must be at least one prospective ACE measured during childhood (< 18 years old) and one mental health outcome measured in adulthood (when subjects are 18 years old or over). The cohort study can include prospective measures of ACEs obtained through official reports of child maltreatment (such as police, court, or child welfare records) that were recorded when the adult participant was a child and are then incorporated into the study through data linkage
Exposure	The ACE(s) was/were reported by anyone who knew the child (I.e., teacher, parent, caregiver, child themselves) or child welfare records or court records
Outcomes	Adult mental health is measured using a continuous or cut-off score approach. Clinical records of adult mental health such as patient records will also be included
Outcomes	The studies do not need to use clinical diagnostic tools for ascertaining mental health conditions but included measurement(s) of mental health outcomes will be validated measures of PTSD, depression, anxiety, suicidal ideation, self-harm, or psychotic-like-experiences. The validation of the tool need not be in the context the study is set in

**Table 2:** Exclusion criteria using the PEO model where applicable

Criteria	Exclusion
Design	Cross-sectional studies
Design	Analyses of interventions will be excluded
Design	Research containing non-empirical work as well as qualitative research and non-original data such as commentaries
Date	Studies published before 1990
Population	Whilst certain “clinical” samples may be included (see inclusion criteria), specific groups that live outside of the general community such as inpatients in psychiatric hospitals or those currently imprisoned will be excluded. This is to allow for more meaningful effect size comparisons between studies as effects in such samples may be overinflated
Exposure	Any study that only includes retrospective measures of ACEs (I.e., the study only includes the reporting of ACEs when the subjects are 18 and over) will be excluded
Exposure	Studies that only use crude measurements of adversity or mental health such as “were you abused as a child” or “have you ever been depressed” without asking about specific symptoms (such as hopelessness for depressed mood) or events (such as being hit for physical abuse)
Exposure	Studies in which the only adversity is a physical trauma without a maltreatment/ abuse/household dysfunction or chronic component (e.g., a car crash)
Outcomes	The studies do not need to use clinical diagnostic tools for ascertaining mental health conditions but included measurement(s) of mental health outcomes will be validated measures of PTSD, depression, anxiety, suicidal ideation, self-harm, or psychotic-like-experiences. The validation of the tool need not be in the context the study is set in

## Information sources

For this review, twelve electronic databases will be searched: Embase, PsycINFO, MEDLINE (Ovid version), and Global Health through the Ovid interface. ProQuest will be used to search Public Affairs Information Service (PAIS), Dissertations and Theses, Sociology Database (including Sociological Abstracts and Social Services Abstracts), PTSDpubs (formerly PILOTS) and ASSIA. CINAHL, WHO Global Index Medicus, and WHO Violence Info will also be searched. The search was conducted throughout the month of June, 2021. An update of the search from June 2021 until March 2023 will also be carried out before the full review to ensure the review includes the most up-to-date research. The search will be limited to publication dates from 1990 onwards and to human subjects in databases that include this limiter. This specific period has been chosen as it aligns with the drafting of the United Nations Convention on the Rights of the Child (UNCRC) by the United Nations [65]. It should be noted that studies published after 1990 that used data from cohorts prior to 1990 will still be eligible if all inclusion criteria are satisfied. This has been decided as the study rationale, research design, research questions, analyses and findings will be interpreted with knowledge from the UNCRC, including a universal definition of when childhood ends and detailed conceptualisations of child protection and maltreatment [66]. The English language specification will be manually screened.

To ensure literature saturation, the authors of this review will email authors of known large cohort studies in the relevant field of research to query whether they have any research that is unfinished/ in the process of being published. Search terms can be found in Appendix 1 and a table of definitions of key concepts can be found in Appendix 2.

## Search strategy

Examples of the search strategies can be found in the Appendices 3, 4, 5, 6, 7 and 8. The search strategy will be altered to account for varying syntax, limiters, and expanders in different databases.

## Data management

Studies identified by the database searches will be extracted and be uploaded to Covidence (a systematic review management software). Before importing search results into Covidence, database citations and abstracts will be exported into Zotero where they will be de-duplicated. Then, references will be transformed into a RIS file format. Once imported to Covidence, duplicates will be checked for and removed again.

## Selection and collection process: screening and extraction

Abstracts and titles will be independently double screened to determine whether the studies meet the inclusion criteria. Next, the remaining papers will be subject to a full-text screen for assessment of inclusion by two reviewers. If necessary, additional information will be sought from the authors of included studies. Any discrepancies in the decision to include a study in the final review will be resolved by team discussion or a third independent reviewer. The final review will include a PRISMA flow diagram documenting the flow of studies throughout the systematic review process.

The final data extracted from the remaining studies will be stored in a spreadsheet on Covidence. The data extracted by reviewers will include:

- General study information (First author, year of study, the format that the information is presented in (e.g., book, article, thesis, conference proceeding)).
- More specific study characteristics (Study location, sample size, sample source (e.g., cohort name), study design (e.g., birth cohort or data linkage), numbers exposed to ACE and outcome).
- Sociodemographic information of participants (gender, age, socio-economic status, ethnicity).
- Information about study variables (measurement/ tool(s) used to collect ACE and mental health data, type of ACEs measured, source of ACEs reporting, type of mental health outcomes measured, age adversity/ mental health was recorded at).
- Information regarding the analysis (metrics, adjustments, results).

## Risk of bias (quality) assessment

Study quality (evaluated in review question 9) will be assessed using the Newcastle–Ottawa Scale for cohort studies and case–control studies (NOS) [67]. This assessment of quality implements a star system based on three overarching domains of study characteristics: Selection of Study Groups, Comparability of Groups and Ascertainment of Exposure/ Outcome. Typically, a maximum of 8 stars can be awarded (A maximum award of 1 star per item within the domains Selection and Exposure and a maximum award of 2 stars for the domain of Comparability) [68]. Two reviewers will independently assess the methodological quality of the included studies and any discrepancies in agreement will be resolved by a third reviewer. However, we will not give each included study an overall quality score or “total star rating”. This is in line with limitations of overall quality scores highlighted in the Cochrane Handbook for Systematic Review of Interventions [69], including a lack of uniformity of quality appraisals across different quality scales being largely attributable to differing conceptualisations of “quality”.

## Data synthesis

A narrative synthesis of included studies will be completed with study information presented in tables and in text. The qualitative discussion will include tabular summaries of the included studies and a discussion of the relationships within and between the studies and will answer review questions 1–4. If enough studies are identified by the database searches and they have enough similarity in design, multi-level meta-analyses will be conducted using the “metafor” [70] package in R to answer review questions 1 and 3–8. The meta-analysis will implement a random-effects model as it is predicted reported effect sizes will vary as a function of exposure, the measurement tools used, and differences in the populations from which the samples are drawn. Specifically, odds ratios (ORs) will be computed in the meta-analysis and when the study outcome is a continuous measure, Hasselblad and Hedges’ [71] method will be used to convert standardised mean differences to log odds ratios. ORs have been cited as a preferred computation for effect size over risk ratio (RR) when computing meta-analyses with binary data (see [72–74]). This is given odds ratios’ symmetry regarding outcome definition and their homogenous, constant nature [75]. The minimum number of studies to permit meta-analyses is two studies per mental health outcome. Again, if enough studies permit, meta-regressions will be conducted in which the moderating effects of the age of adversity onset, country, types of adversity, publication status, and duration of follow-up period will be explored.

I^2^ will be used to assess statistical heterogeneity. It was originally intended to be independent of the number of studies (unlike Cochran’s Q) and has been regularly used in Cochrane reviews [76]. However, it should be noted some research suggests I^2^ can still be biased in small meta-analyses [77].

## Meta bias(es)

The possibility of publication/ dissemination bias in the identified studies will be explored. Publication bias will be identified and corrected by first using the “trim and fill” method [78] which will be conducted for each outcome in the meta-analysis. This procedure will help detect and correct any asymmetry in the funnel plots. The Egger bias test will be computed for further examination of funnel plot asymmetry [79].

## Discussion

The purpose of this review is to systematically investigate the existence and strength of association between ACEs and adult mental health outcomes in prospective longitudinal studies with a focus on the mental health outcomes anxiety, depression, PTSD, self-harm, suicidal ideation, and psychotic-like experiences.

First, by exploring associations between ACEs and key mental health outcomes, we aim to evaluate the importance of identifying prospectively measured individual ACEs and cumulative ACE scores as risk markers for later poor mental health outcomes in adults [45]. Second, by exploring how ACEs relate to different mental health outcomes, we may assist in the future prioritisation of specific preventative mental health interventions in ACE-exposed populations. Third, we will also evaluate whether studies in the field of childhood adversity are affected by publication bias. This will provide further insight as to whether the included published studies are a representative sample of available evidence of the longitudinal associations between ACEs and adult mental health. Lastly, this review may have further implications for ACEs research such as identifying methodological weaknesses and knowledge gaps in literature that can be addressed in future primary studies. For example, we may be able to tell what ACEs and mental health outcomes are under-researched and whether there are regions of the world that are under-represented or missing from the literature.

The authors acknowledge the risk of bias that results from being unable to include studies not readily available in English. Whilst this decision was made due to resource constraints, authors may miss high-quality studies and key data [80]. We must also consider limitations associated with the use of official records (e.g., child protective service records or court cases) to obtain information about ACE exposure in prospective ACE studies. Official records are more likely to include only the most severe cases of childhood adversity and are more likely to document ACEs that happened chronically or earlier in life [81]. They subsequently miss childhood experiences that may not require official child protective services record such as childhood bullying or parental divorce, but that may still be significantly associated with poor outcomes [82, 83]. Furthermore, prospectively measured ACEs may also be vulnerable to under-reporting due to substantiation bias, report bias, investigation bias, and issues relating to stigma and secrecy [84–86]. Despite the limitations outlined, prospective measures of ACEs provide valuable information about temporal patterning of ACEs and later-life mental ill-health.

In conclusion, studies exploring longitudinal associations between ACEs and adult mental health outcomes have already been synthesised, but this review aims to expand the existing systematic review methodological and analytical approaches. We aim to offer valuable insights about the associations between ACEs and mental health outcomes, their moderators, the quality of longitudinal ACEs studies, specific methodological weaknesses and knowledge gaps that may influence future research directions such as targeting under-researched locations, ACEs, and mental health outcomes.

### Supplementary Information

**Additional file 1.** PRISMA-P 2015 Checklist.

## Data Availability

Not applicable.

## Appendix 1

Table of Search Terms

**Table:**

Domain	Search	Search Term
**Population**	S1	Child*
	S2	Infan*
	S3	Teen*
	S4	Adolescen*
	S5	School-age*
	S6	School Age*
	S7	Toddler*
	S8	Baby
	S9	Babies
	S10	Newborn*
	S11	Kid*
	S12	Minor*
	S13	Preschool*
	S14	Pre-School*
	S15	Underage*
	S16	Under-age*
	S17	Juvenil*
	S18	Perinatal*
	S19	Youth*
	S20	Young Pe*
	S21	S1 OR S2 OR S3 OR S4 OR S5 OR S6 OR S7 OR S8 OR S9 OR S10 OR S11 OR S12 OR S13 OR S14 OR S15 OR S16 OR S17 OR S18 OR S19 OR S20
	S22	“Adverse Childhood Experiences”
**Interest**	S23	Advers*
Adverse childhood experiences	S24	ACE*
	S25	Trauma*
	S26	Maltreat*
	S27	Violence
	S28	Abuse*
	S29	Parent* Substance
	S30	Parent* Alcohol
	S31	Parent* Illicit drug*
	S32	Parent* Prescription drug*
	S33	Parent* Drug*
	S34	Parent* Cannabis
	S35	Parent* Cocaine
	S36	Parent* Heroin
	S37	Parent* Separation
	S38	Parent* Divorce
	S39	Parent* Break*
	S40	Parent* mental*
	S41	Parent* death
	S42	Parent* morbidit*
	S43	Parent* ill*
	S44	Parent* Disorder*
	S45	Terrori*
	S46	Coup*
	S47	Riot*
	S48	Revolution*
	S49	Household Dysfunction
	S50	Neglect*
	S51	Bully*
	S52	Bullie*
	S53	Conflict
	S54	Exploit*
	S55	Polyvictimi*
	S56	Victimi*
	S57	Peer Violence
	S58	Peer-violence
	S59	Marriage
	S60	War*
	S61	Assault*
	S62	Prostitut*
	S63	Sex-work
	S64	Sex Work
	S65	Traffick*
	S66	Displace*
	S67	S22 OR S23 OR S24 OR S25 OR S26 OR S27 OR S28 OR S29 OR S30 OR S31 OR S32 OR S33 OR S34 OR S35 OR S36 OR S37 OR S38 OR S39 OR S40 OR S41 OR S42 OR S43 OR S44 OR S45 OR S46 OR S47 OR S48 OR S49 OR S50 OR S51 OR S52 OR S53 OR S54 OR S55 OR S56 OR S57 OR S58 OR S59 OR S60 OR S61 OR S62 OR S63 OR S64 OR S65 OR S66
**Outcome**	S68	Mental Health
Mental Health	S69	Mental* Ill*
	S70	Mental disorder*
	S71	Anxiety
	S72	Panic Disorder
	S73	Obsessive Compulsive Disorder
	S74	Social phobia
	S75	Post-traumatic stress
	S76	Post Traumatic stress
	S77	Acute stress
	S78	Suicid*
	S79	Self-harm
	S80	Self Harm*
	S81	Self-injur*
	S82	Self Injur*
	S83	Self-poison*
	S84	Self Poison*
	S85	Self-punish*
	S86	Self Punish*
	S87	Psycho*
	S88	Schizo*
	S89	Delusion*
	S90	Hallucination*
	S91	Paranoi*
	S92	Magical Thinking
	S93	abnormal*
	S94	Paranormal*
	S95	Odd beliefs
	S96	Depress*
	S97	S68 OR S69 OR S70 OR S71 OR S72 OR S73 OR S74 OR S75 OR S76 OR S77 OR S78 OR S79 OR S80 OR S81 OR S82 OR S83 OR S84 OR S85 OR S86 OR S87 OR S88 OR S89 OR S90 OR S91 OR S92 OR S93 OR S94 OR S95 OR S96
**Design**	S98	Prospective
Longitudinal Designs	S99	Longitudinal*
	S100	Cohort
	S101	Panel
	S102	S98 OR S99 OR S100 OR S101
**Population and Interest and Outcome and Design**	**S103**	**S21 AND S67 AND S97 AND S102**

## Appendix 2

Table of Definitions of Key Concepts

**Table:**

Children/ Childhood/ Child	We will use the United Nations Convention on the Rights of the Child (UNCRC) [65] definition of a child: “a child means every human being below the age of 18 years”
Adverse Childhood Experiences	We will use the Centre for Disease Control and Prevention (CDC) (3: p.7) definition of adverse childhood experiences as “potentially traumatic events that occur in childhood (0–17 years)”
ACE: Child Physical Abuse	We will use the term “child physical abuse” to also refer to child physical violence We will use the World Health Organisation’s [87] definition of child physical abuse: “Physical abuse of a child is defined as those acts of commission by a caregiver that cause actual physical harm or have the potential for harm.” The actions inflicted on a child may include: Hitting (with hands or objects), pushing, grabbing, slapping, throwing something at the child, kicking, shaking, burning/ scalding, biting, scratching, breaking bones, drowning, or poisoning
ACE: Child Emotional Abuse	We will use “child emotional abuse” to also describe psychological abuse and verbal abuse Emotional abuse will be defined by the World Health Organisation’s [87] definition of child emotional abuse: “Emotional abuse includes the failure of a caregiver to provide an appropriate and supportive environment and includes acts that have an adverse effect on the emotional health and development of a child.” This may include parents or caregivers: Frequently insulting or criticising the child, humiliation, threatening a child, blaming, and scapegoating, making a child perform degrading acts, not allowing a child to have friends, manipulation of a child, ignoring a child, being absent
ACE: Child Sexual Abuse	We will use the term “child sexual abuse” to also refer to child sexual exploitation, child prostitution, and child sex work We will use the World Health Organisation’s [88] definition of child sexual abuse: “The involvement of a child in sexual activity that he or she does not fully comprehend and is unable to give informed consent to, or for which the child is not developmentally prepared, or else that violate the laws or social taboos of society. Child sexual abuse is evidenced by this activity between a child and an adult or another child who by age or development is in a relationship of responsibility, trust or power, the activity being intended to gratify or satisfy the needs of the other person” Types of sexual abuse can include: Sexual touching by an adult or peer of any part of a child’s body, a child being forced or tricked into touching an adult or peer’s body in a sexual way, an adult or peer attempting oral, anal, or vaginal intercourse, an adult or peer that has oral, anal, or vaginal intercourse with a child Our definition also includes non-contact sexual abuse such as: An adult or peer exposing themselves, an adult or peer showing pornography to a child, an adult or peer making a child masturbate, an adult or peer forcing a child to take/ share/ view child abuse images or videos, an adult or peer taking part in sexual conversations with a child (face to face or online)
ACE: Child Neglect	We will use the term “child neglect” to refer to medical neglect, educational neglect, emotional neglect, and physical neglect We will use the World Health Organisation’s [87] definition of neglect: “Neglect refers to the failure of a parent to provide for the development of the child – where the parent is in a position to do so – in one or more of the following areas: health, education, emotional development, nutrition, shelter and safe living conditions. Neglect is thus distinguished from circumstances of poverty in that neglect can occur only in cases where reasonable resources are available to the family or caregiver.”
ACE: Peer Victimisation	We will use the term “peer victimisation” as an umbrella term to also describe peer violence or bullying Peer victimisation may include: Name calling, other verbal threats and insults, physical assault (hitting, punching, slapping, throwing objects at the victim), and bullying on electronic platforms such as social media, private texts, or emails
ACE: Exposure to Domestic Violence	We will use the term “exposure to domestic violence” to also refer to exposure to domestic abuse or exposure to intimate partner violence between adults in the home We will define exposure to domestic violence as a child witnessing or overhearing domestic violence between two or more adults in their household. Domestic violence is defined using the United Nations’ definition: “Domestic abuse, also called "domestic violence" or "intimate partner violence", can be defined as a pattern of behaviour in any relationship that is used to gain or maintain power and control over an intimate partner.” Domestic abuse between adults that a child may witness or overhear is “physical, sexual, emotional, economic, or psychological actions or threats of actions that influence another person. This includes any behaviours that frighten, intimidate, terrorize, manipulate, hurt, humiliate, blame, injure, or wound someone” [89]
ACE: Parental or caregiver morbidity or mortality	We will use the term “parental/ caregiver mortality” to also refer to parental/caregiver death and will use the term “parental/ caregiver morbidity” to refer to parental/caregiver illness or sickness Mortality refers to the death of a parent/ caregiver prior to a child’s 18^th^ birthday. Parental/ caregiver morbidity refers to “the state of being symptomatic or unhealthy for a disease or condition” [90]. In this case, parental/ caregiver morbidity will not refer to mental illness due to this being a distinct adverse childhood experience category
ACE: Parental or caregiver mental illness	We will use the term “parental or caregiver mental illness” to also refer to parental/caregiver mental sickness, poor mental health, mental disorder, or mental ill-health We will use the following definition of mental illness from Chadda ([91]: p.12): “Mental Illness refers to a chronic disturbance of mood, thought, perception, orientation or memory, which causes significant impairment in a person's behaviour, judgment, and ability to recognize reality or impairs the persons’ ability to meet the demands and activities of daily life.”
ACE: Parental or caregiver substance misuse	We will use the term “parental or caregiver substance misuse” to also refer to injected drug use, heavy alcohol use, heavy drug use, drug misuse, alcohol misuse, problematic drug or alcohol use, substance abuse, heroin, crack cocaine, ecstasy, valium, GHB or cannabis use, prescription drug abuse or misuse We will use the NSPCC’s [92] definition of parental or caregiver substance misuse: “Parental substance misuse’ is the long-term misuse of drugs and/or alcohol by a parent or carer This includes parents and carers who: • consume harmful amounts of alcohol (for example if their drinking is leading to alcohol-related health problems or accidents) • are dependent on alcohol • use drugs regularly and excessively • are dependent on drugs It also includes parents [and carer’s] who are not able to supervise their children appropriately because of their substance use.”
ACE: Parental/ caregiver Separation	We will use the term “parental/ caregiver separation” to refer to parental/ caregiver break-up, breakdown, or divorce We will define parental separation as any relationship breakdown between parents or caregivers with no specificity on marital status or whether the separation was acrimonious or peaceful
ACE: Community Violence	We will use the term “community violence” to also refer to group violence and gang violence We have defined community violence using The National Child Traumatic Stress Network’s [93] definition: “Exposure to intentional acts of interpersonal violence committed in public areas by individuals who are not intimately related to the victim” The acts of violence may include witnessing or involvement in shooting, gang fights, civil wars, stabbing or threatening with a gun
ACE: Collective Violence	We will use the term “collective violence” as a term to also refer to, exposure to state-sanctioned violence, terrorism, rebellions, wars, terrorism, coups, revolutions, rioting We will use the World Health Organisation’s [94] definition of collective violence: “Collective violence includes violent conflicts between nations and groups, state and group terrorism, rape as a weapon of war, the movement of large numbers of people displaced from their homes, and gang warfare.”
Depression	We will use the term “depression” to also define depressive symptoms, major depression, depressive disorders, mood disorder and depressed affect. We will also consider studies to include the outcome “depression” if they measure any symptoms of depression that are specified in the “Depressive Disorders” section of the ICD-11 [95]
Anxiety	We will use the term “anxiety” to also define frequent anxiety, generalised anxiety disorder, obsessive compulsive disorder, panic disorder, social phobia, and social anxiety disorder. We will also consider studies to include the outcome “anxiety” if they measure any symptoms of anxiety that are specified in the “Anxiety or fear-related disorders” section of the ICD-11 [95]
Post-Traumatic Stress Disorder (PTSD)	We will use the term “PTSD” to also define complex PTSD, comorbid PTSD, uncomplicated PTSD, and acute stress disorder. We will also consider studies to include the outcome “PTSD” if they measure any symptoms of PTSD that are specified in the “Post traumatic stress disorder” or “Complex post-traumatic stress disorder” sections of the ICD-11 [95]
Suicidal	We will use the term “suicidal” to also define suicidal thoughts, suicidal ideation, planning suicide, attempting suicide, feeling suicidal, considering suicide, and suicidal tendencies. We will also consider studies to include the outcome “suicidal ideation” if they include measurement of the symptoms or signs of suicidality specified in the “Suicidal behaviour” or “Suicide attempt” sections of the ICD-11 [95]
Self-harm	We will use the term “self-harm” to also define self-injury, self-poisoning, self-punishment, and non-suicidal self-injury. We will also consider studies to include the outcome “self-harm” if they include measurement of the symptoms or signs of self-harm specified in the “Intentional self-harm” or “Non-suicidal self-injury” sections of the ICD-11 [95]
Psychotic-like experiences	We will use the term “psychotic-like experiences” to also refer to psychotic disorders, psychosis, schizotypal traits, schizophrenia, schizoaffective disorder, psychotic symptoms, delusions, hallucinations, magical thinking, persecutory ideas, bizarre experiences, perceptual abnormalities, paranormal beliefs, odd beliefs, paranoia. We will also consider studies to include the outcome “psychotic-like experiences” if they measure any symptoms of psychotic-like experiences that are defined in the “Schizophrenia or other primary psychotic disorders” section of the ICD-11 [95]. The term “psychotic-like” was preferred over “psychosis-like” in line with a large body of literature in which this is the predominant phrasing to describe subthreshold psychotic symptoms in the general population

## Appendix 3

Ovid Search Strategy

SEARCH 1- Embase (1980 to 2021 Week 19); Global Health (1973 to 2021 Week 19); APA PsycInfo (1806 to 2021 Week 19); and Ovid MEDLINE® and Epub Ahead of Print, In-Process, In-Data-Review & Other Non-Indexed Citations and Daily (1946 to 2021 Week 19)

10-June-21

**Table:**

Domain	String #	Search Term	Limiters Applied	Results
POPULATION (Children)	1	(Child* OR Infan* OR Teen* OR Adolescen* OR School-age* OR "School Age*" OR Toddler* OR Baby OR Babies OR Newborn* OR Kid* OR Minor* OR Preschool* OR Pre-School* OR Underage* OR Under-age* OR Juvenile* OR Perinatal* OR Youth* OR "Young Pe*").m_titl		3,936,707
	2	#1	Humans [Limit not valid in Global Health or APA Psyc Info] Yr = “1990- Current”	2,864,040
INTEREST (Adverse Childhood Experiences: excluding parental adversities)	3	(Advers* OR ACE* OR "Adverse Childhood Experience*" OR Trauma* OR Violence OR Abuse* OR Terrori* OR Coup* OR Riot* OR Revolution OR "Household Dysfunction*" OR Neglect* OR Bully* OR Bullie* OR Conflict* OR Exploit* OR Polyvictimi* OR Victimi* OR "Peer Violence" OR Peer-Violence OR Marriage OR War* OR Assault* OR Prostitut* OR Sex-Work* OR "Sex Work*" OR Traffick* OR Displace*).m_titl		1,847,836
	4	#3	Humans [Limit not valid in Global Health or APA PsycInfo] Yr = “1990- Current”	1,104,923
INTEREST (Adverse Childhood Experiences: exclusively parental adversities)	5	(Parent* adj1 (Substance* or Alcohol* or "Illicit Drug*" or "Prescription Drug*" or Drug* or Cannabis or Cocaine or Heroin or Separat* or Divorce* or Break* or Mental* or Disorder* or Death or Morbidit* or Ill*)).m_titl		6701
	6	#5	Humans [Limit not valid in Global Health or APA PsycInfo] Yr = “1990- Current”	5071
OUTCOME (Adult Mental Health)	7	(“Mental Health” OR "Mental* Ill*" OR "Mental Disorder*" OR Anxiety OR "Panic Disorder" OR "Obsessive Compulsive Disorder" OR "Social Phobia" OR "Post-Traumatic Stress" OR "Post Traumatic Stress" OR "Acute Stress" OR Suicid* OR Self-Harm* OR Self-Injur* OR Self-Poison* OR Self-Punish* OR "Self Harm*" OR "Self Injur*" OR "Self Poison*" OR "Self Punish*" OR Psycho* OR Schizo* OR Delusion* OR Hallucination* OR Paranoi* OR "Magical Thinking" OR Abnormal* OR Paranormal* OR "Odd Beliefs*” OR Depress*).m_titl		2,342,919
	8	#7	Humans [Limit not valid in Global Health or APA PsycInfo] Yr = “1990- Current”	1,690,597
DESIGN	9	(Longitudinal* or Cohort* or Panel* or Prospective*).mp. [mp = ti, ab, hw, tn, ot, dm, mf, dv, kw, fx, dq, tc, id, tm, mh, sh, bt, cc, nm, kf, ox, px, rx, an, ui, sy]		555,223
	10	#9	Humans [Limit not valid in Global Health or APA Psyc Info] Yr = “1990- Current”	
ALL DOMAINS	11	2 AND (4 OR 6) AND 8 AND 10		4176
ALL DOMAINS (DE-DUPLICATED)				1193

## Appendix 4

CINAHL Search Strategy (Through EBSCOhost)

SEARCH 2- CINAHL Plus

Search Modes – Boolean/ Phrase

Search Expanders – Apply equivalent subjects

10-June-21

**Table:**

Domain	String #	Search Term	Limiters Applied	Results
POPULATION (Children)	1	TI Child* OR Infan* OR Teen* OR Adolescen* OR School-age* OR "School Age*" OR Toddler* OR Baby OR Babies OR Newborn* OR Kid* OR Minor* OR Preschool* OR Pre-School* OR Underage* OR Under-age* OR Juvenile* OR Perinatal* OR Youth* OR "Young Pe*”		554,142
	2	#1	Humans Yr = “1990- Current”	273,338
INTEREST (Adverse Childhood Experiences: excluding parental adversities)	3	TI Advers* OR ACE* OR "Adverse Childhood Experience*" OR Trauma* OR Violence OR Abuse* OR Terrori* OR Coup* OR Riot* OR Revolution OR "Household Dysfunction*" OR Neglect* OR Bully* OR Bullie* OR Conflict* OR Exploit* OR Polyvictimi* OR Victimi* OR "Peer Violence" OR Peer-Violence OR Marriage OR War* OR Assault* OR Prostitut* OR Sex-Work* OR "Sex Work*" OR Traffick* OR Displace*		228,670
	4	#3	Humans Yr = “1990- Current”	92,665
INTEREST (Adverse Childhood Experiences: exclusively parental adversities)	5	TI "Parent* Substance*" or "Parent* Alcohol*" or "Parent* Illicit Drug*" or "Parent* Prescription Drug*" or "Parent* Drug*" or "Parent* Cannabis" or "Parent* Cocaine" or "Parent* Heroin" or "Parent* Separat*" or "Parent* Divorce*" or "Parent* Break*" or "Parent* Mental*" or "Parent* Disorder*" or "Parent* Death" or "Parent* Morbidit*" or "Parent* Ill*"		835
	6	#5	Humans Yr = “1990- Current”	561
OUTCOME (Adult Mental Health)	7	TI “Mental Health” OR "Mental* Ill*" OR "Mental Disorder*" OR Anxiety OR "Panic Disorder" OR "Obsessive Compulsive Disorder" OR "Social Phobia" OR "Post-Traumatic Stress" OR "Post Traumatic Stress" OR "Acute Stress" OR Suicid* OR Self-Harm* OR Self-Injur* OR Self-Poison* OR Self-Punish* OR "Self Harm*" OR "Self Injur*" OR "Self Poison*" OR "Self Punish*" OR Psycho* OR Schizo* OR Delusion* OR Hallucination* OR Paranoi* OR "Magical Thinking" OR Abnormal* OR Paranormal* OR "Odd Beliefs*” OR Depress*		289,683
	8	#7	Humans Yr = “1990- Current”	145,955
DESIGN	9	TX Longitudinal* or Cohort* or Panel* or Prospective*		758,949
	10	#9	Humans Yr = “1990- Current”	549,797
ALL DOMAINS	11	2 AND (4 OR 6) AND 8 AND 10		709

## Appendix 5

ProQuest Dissertations and Theses

SEARCH 3- ProQuest Dissertations and Theses

3-June-21

**Table:**

Domain	String #	Search Term	Limiters Applied	Results
POPULATION (Children)	1	Ti(Child* OR Infan* OR Teen* OR Adolescen* OR School-age* OR "School Age*" OR Toddler* OR Baby OR Babies OR Newborn* OR Kid* OR Minor* OR Preschool* OR Pre-School* OR Underage* OR Under-age* OR Juvenile* OR Perinatal* OR Youth* OR "Young Pe*”)		9,148,758
	2	#1	Dissertations & Theses Yr = “1990- Current”	79,030
INTEREST (Adverse Childhood Experiences: excluding parental adversities)	3	Ti(Advers* OR ACE* OR "Adverse Childhood Experience*" OR Trauma* OR Violence OR Abuse* OR Terrori* OR Coup* OR Riot* OR Revolution OR "Household Dysfunction*" OR Neglect* OR Bully* OR Bullie* OR Conflict* OR Exploit* OR Polyvictimi* OR Victimi* OR "Peer Violence" OR Peer-Violence OR Marriage OR War* OR Assault* OR Prostitut* OR Sex-Work* OR "Sex Work*" OR Traffick* OR Displace*)		19,767,404
	4	#3	Dissertations & Theses Yr = “1990- Current”	109,096
INTEREST (Adverse Childhood Experiences: exclusively parental adversities)	5	Ti("Parent* Substance*" or "Parent* Alcohol*" or "Parent* Illicit Drug*" or "Parent* Prescription Drug*" or "Parent* Drug*" or "Parent* Cannabis" or "Parent* Cocaine" or "Parent* Heroin" or "Parent* Separat*" or "Parent* Divorce*" or "Parent* Break*" or "Parent* Mental*" or "Parent* Disorder*" or "Parent* Death" or "Parent* Morbidit*" or "Parent* Ill*")		7037
	6	#5	Dissertations & Theses Yr = “1990- Current”	576
OUTCOME (Adult Mental Health)	7	Ti(“Mental Health” OR "Mental* Ill*" OR "Mental Disorder*" OR Anxiety OR "Panic Disorder" OR "Obsessive Compulsive Disorder" OR "Social Phobia" OR "Post-Traumatic Stress" OR "Post Traumatic Stress" OR "Acute Stress" OR Suicid* OR Self-Harm* OR Self-Injur* OR Self-Poison* OR Self-Punish* OR "Self Harm*" OR "Self Injur*" OR "Self Poison*" OR "Self Punish*" OR Psycho* OR Schizo* OR Delusion* OR Hallucination* OR Paranoi* OR "Magical Thinking" OR Abnormal* OR Paranormal* OR "Odd Beliefs*” OR Depress*)		2,053,836
	8	#7	Dissertations & Theses Yr = “1990- Current”	48,963
DESIGN	9	TX(Longitudinal* or Cohort* or Panel* or Prospective*)		3,009,142
	10	#9	Dissertations & Theses Yr = “1990- Current”	130,979
ALL DOMAINS	11	2 AND (4 OR 6) AND 8 AND 10		110

## Appendix 6

Applied Social Sciences Index and Abstracts Search Strategy

SEARCH 5- Applied Social Sciences Index and Abstracts (ProQuest)

3-June-21

**Table:**

Domain	String #	Search Term	Limiters Applied	Results
POPULATION (Children)	1	Ti(Child* OR Infan* OR Teen* OR Adolescen* OR School-age* OR "School Age*" OR Toddler* OR Baby OR Babies OR Newborn* OR Kid* OR Minor* OR Preschool* OR Pre-School* OR Underage* OR Under-age* OR Juvenile* OR Perinatal* OR Youth* OR "Young Pe*”)		185,424
	2	#1	Yr = “1990- Current”	177,827
INTEREST (Adverse Childhood Experiences: excluding parental adversities)	3	Ti(Advers* OR ACE* OR "Adverse Childhood Experience*" OR Trauma* OR Violence OR Abuse* OR Terrori* OR Coup* OR Riot* OR Revolution OR "Household Dysfunction*" OR Neglect* OR Bully* OR Bullie* OR Conflict* OR Exploit* OR Polyvictimi* OR Victimi* OR "Peer Violence" OR Peer-Violence OR Marriage OR War* OR Assault* OR Prostitut* OR Sex-Work* OR "Sex Work*" OR Traffick* OR Displace*)		73,910
	4	#3	Yr = “1990- Current”	70,553
INTEREST (Adverse Childhood Experiences: exclusively parental adversities)	5	Ti("Parent* Substance*" or "Parent* Alcohol*" or "Parent* Illicit Drug*" or "Parent* Prescription Drug*" or "Parent* Drug*" or "Parent* Cannabis" or "Parent* Cocaine" or "Parent* Heroin" or "Parent* Separat*" or "Parent* Divorce*" or "Parent* Break*" or "Parent* Mental*" or "Parent* Disorder*" or "Parent* Death" or "Parent* Morbidit*" or "Parent* Ill*")		937
	6	#5	Yr = “1990- Current”	897
OUTCOME (Adult Mental Health)	7	Ti(“Mental Health” OR "Mental* Ill*" OR "Mental Disorder*" OR Anxiety OR "Panic Disorder" OR "Obsessive Compulsive Disorder" OR "Social Phobia" OR "Post-Traumatic Stress" OR "Post Traumatic Stress" OR "Acute Stress" OR Suicid* OR Self-Harm* OR Self-Injur* OR Self-Poison* OR Self-Punish* OR "Self Harm*" OR "Self Injur*" OR "Self Poison*" OR "Self Punish*" OR Psycho* OR Schizo* OR Delusion* OR Hallucination* OR Paranoi* OR "Magical Thinking" OR Abnormal* OR Paranormal* OR "Odd Beliefs*” OR Depress*)		100,212
	8	#7	Yr = “1990- Current”	97,021
DESIGN	9	Ab(Longitudinal* or Cohort* or Panel* or Prospective*)		78,636
	10	#9	Yr = “1990- Current”	77,405
ALL DOMAINS	11	2 AND (4 OR 6) AND 8 AND 10		326

## Appendix 7

ProQuest PTSDpubs

SEARCH 7- ProQuest Dissertations and Theses

3-June-21

**Table:**

Domain	String #	Search Term	Limiters Applied	Results
POPULATION (Children)	1	Ti(Child* OR Infan* OR Teen* OR Adolescen* OR School-age* OR "School Age*" OR Toddler* OR Baby OR Babies OR Newborn* OR Kid* OR Minor* OR Preschool* OR Pre-School* OR Underage* OR Under-age* OR Juvenile* OR Perinatal* OR Youth* OR "Young Pe*”)		11,383
	2	#1	Yr = “1990- Current”	10,873
INTEREST (Adverse Childhood Experiences: excluding parental adversities)	3	Ti(Advers* OR ACE* OR "Adverse Childhood Experience*" OR Trauma* OR Violence OR Abuse* OR Terrori* OR Coup* OR Riot* OR Revolution OR "Household Dysfunction*" OR Neglect* OR Bully* OR Bullie* OR Conflict* OR Exploit* OR Polyvictimi* OR Victimi* OR "Peer Violence" OR Peer-Violence OR Marriage OR War* OR Assault* OR Prostitut* OR Sex-Work* OR "Sex Work*" OR Traffick* OR Displace*)		27,276
	4	#3	Yr = “1990- Current”	25,669
INTEREST (Adverse Childhood Experiences: exclusively parental adversities)	5	Ti("Parent* Substance*" or "Parent* Alcohol*" or "Parent* Illicit Drug*" or "Parent* Prescription Drug*" or "Parent* Drug*" or "Parent* Cannabis" or "Parent* Cocaine" or "Parent* Heroin" or "Parent* Separat*" or "Parent* Divorce*" or "Parent* Break*" or "Parent* Mental*" or "Parent* Disorder*" or "Parent* Death" or "Parent* Morbidit*" or "Parent* Ill*")		43
	6	#5	Yr = “1990- Current”	42
OUTCOME (Adult Mental Health)	7	Ti(“Mental Health” OR "Mental* Ill*" OR "Mental Disorder*" OR Anxiety OR "Panic Disorder" OR "Obsessive Compulsive Disorder" OR "Social Phobia" OR "Post-Traumatic Stress" OR "Post Traumatic Stress" OR "Acute Stress" OR Suicid* OR Self-Harm* OR Self-Injur* OR Self-Poison* OR Self-Punish* OR "Self Harm*" OR "Self Injur*" OR "Self Poison*" OR "Self Punish*" OR Psycho* OR Schizo* OR Delusion* OR Hallucination* OR Paranoi* OR "Magical Thinking" OR Abnormal* OR Paranormal* OR "Odd Beliefs*” OR Depress*)		14,199
	8	#7	Yr = “1990- Current”	13,440
DESIGN	9	TX Longitudinal* or Cohort* or Panel* or Prospective*		4939
	10	#9	Yr = “1990- Current”	4850
ALL DOMAINS	11	2 AND (4 OR 6) AND 8 AND 10		141

## Appendix 8

World Health Organisation Global Index Medicus Search Strategy

SEARCH 8- WHO GMI

8-June-21

**Table:**

Domain	String #	Search Term	Limiters Applied	Results
POPULATION (Children)	1	(tw:(Child* OR Infan* OR Teen* OR Adolescen* OR "School-age" OR Toddler* OR Baby OR Babies OR Newborn* OR Kid* OR Minor* OR Preschool* OR "Pre School" OR Pre-School* OR Underage* OR Under-age* OR "Under Age" OR Juvenile* OR Youth* OR Young-people OR "Young people"))		319,171
	2	#1	Yr = “1990- Current”	291,438
INTEREST (Adverse Childhood Experiences: excluding parental adversities)	3	(tw:(Advers* OR ACE* OR "Adverse Childhood Experience*" OR Trauma* OR Violence OR Abuse* OR Terrori* OR Coup* OR Riot* OR Revolution OR "Household Dysfunction*" OR Neglect* OR Bully* OR Bullie* OR Conflict* OR Exploit* OR Polyvictimi* OR Victimi* OR "Peer Violence" OR Peer-Violence OR Marriage OR War* OR Assault* OR Prostitut* OR Sex-Work* OR "Sex Work*" OR Traffick* OR Displace*))		279,181
	4	#3	Yr = “1990- Current”	268,414
INTEREST (Adverse Childhood Experiences: exclusively parental adversities)	5	(tw:("Parental Substance" or "Parental Alcohol" or "Parental Illicit Drug" or "Parental Prescription Drug" or "Parental Drug" or "Parental Cannabis" or "Parental Cocaine" or "Parental Heroin" or "Parental Separation" or "Parental Divorce" or "Parental Break up" OR "Parental Break Down" or "Parental Mental" or "Parental Disorder" or "Parental Death" or "Parental Morbidity" or "Parental Illness"))		725
	6	#5	Yr = “1990- Current”	709
OUTCOME (Adult Mental Health)	7	(tw:(“Mental Health” OR "Mental* Ill*" OR "Mental Disorder*" OR Anxiety OR "Panic Disorder" OR "Obsessive Compulsive Disorder" OR "Social Phobia" OR "Post-Traumatic Stress" OR "Post Traumatic Stress" OR "Acute Stress" OR Suicid* OR Self-Harm* OR Self-Injur* OR Self-Poison* OR Self-Punish* OR "Self Harm*" OR "Self Injur*" OR "Self Poison*" OR "Self Punish*" OR Psycho* OR Schizo* OR Delusion* OR Hallucination* OR Paranoi* OR "Magical Thinking" OR Abnormal* OR Paranormal* OR "Odd Beliefs*” OR Depress*)		55,332
	8	#7	Yr = “1990- Current”	54,137
DESIGN	9	(tw:(Longitudinal* or Cohort* or Panel* or Prospective*))		74,271
	10	#9	Yr = “1990- Current”	73,165
ALL DOMAINS	11	2 AND (4 OR 6) AND 8 AND 10		333

## Abbreviations

ACEs: Adverse Childhood Experiences
ASSIA: Applied Social Sciences Index and Abstracts
CDC: Centres for Disease Control and Prevention
HAC: Health Appraisal Centre
NOS: Newcastle–Ottawa Scale
NSPCC: National Society for the Prevention of Cruelty to Children
OR: Odds Ratio
PAIS: Public Affairs Information Service
PILOTS: Published International Literature on Traumatic Stress
PRISMA-P: Preferred Reporting Items for Systematic Review and Meta-Analysis Protocols
PRISMA: Preferred Reporting Items for Systematic Review and Meta-Analysis
PTSD: Post-traumatic Stress Disorder
RR: Risk Ratio
UNCRC: United Nations Convention on the Rights of the Child
WHO: World Health Organisation
